# Paternal X inactivation does not correlate with X chromosome evolutionary strata in marsupials

**DOI:** 10.1186/s12862-014-0267-z

**Published:** 2014-12-24

**Authors:** Claudia L Rodríguez-Delgado, Shafagh A Waters, Paul D Waters

**Affiliations:** Division of Evolution, Ecology and Genetics, Research School of Biology, The Australian National University, Canberra, ACT 0200 Australia; School of Biotechnology and Biomolecular Sciences, Faculty of Science, University of New South Wales, Sydney, NSW 2052, Australia

**Keywords:** X inactivation, Marsupial, Imprinting, X chromosome, Escape, RNA-seq, SNP, Evolution, Strata

## Abstract

**Background:**

X chromosome inactivation is the transcriptional silencing of one X chromosome in the somatic cells of female mammals. In eutherian mammals (e.g. humans) one of the two X chromosomes is randomly chosen for silencing, with about 15% (usually in younger evolutionary strata of the X chromosome) of genes escaping this silencing. In contrast, in the distantly related marsupial mammals the paternally derived X is silenced, although not as completely as the eutherian X. A chromosome wide examination of X inactivation, using RNA-seq, was recently undertaken in grey short-tailed opossum (*Monodelphis domestica*) brain and extraembryonic tissues. However, no such study has been conduced in Australian marsupials, which diverged from their American cousins ~80 million years ago, leaving a large gap in our understanding of marsupial X inactivation.

**Results:**

We used RNA-seq data from blood or liver of a family (mother, father and daughter) of tammar wallabies (*Macropus eugenii*), which in conjunction with available genome sequence from the mother and father, permitted genotyping of 42 expressed heterozygous SNPs on the daughter’s X. These 42 SNPs represented 34 X loci, of which 68% (23 of the 34) were confirmed as inactivated on the paternally derived X in the daughter’s liver; the remaining 11 X loci escaped inactivation. Seven of the wallaby loci sampled were part of the old X evolutionary stratum, of which three escaped inactivation. Three loci were classified as part of the newer X stratum, of which two escaped inactivation. A meta-analysis of previously published opossum X inactivation data revealed that 5 of 52 genes in the old X stratum escaped inactivation.

**Conclusions:**

We demonstrate that chromosome wide inactivation of the paternal X is common to an Australian marsupial representative, but that there is more escape from inactivation than reported for opossum (32% v 14%). We also provide evidence that, unlike the human X chromosome, the location of loci within the oldest evolutionary stratum on the marsupial X does not correlate with their probability of escape from inactivation.

**Electronic supplementary material:**

The online version of this article (doi:10.1186/s12862-014-0267-z) contains supplementary material, which is available to authorized users.

## Background

Therian (i.e. eutherian and marsupial) mammals have a XX female/XY male sex chromosome system, or some simple variant of it. The X and Y chromosomes evolved from an ordinary pair of autosomes via a process of gene loss on the Y [1], which resulted in an imbalance of X gene dosage with the autosomes (1X: 2A gene dosage) in males. It was proposed that to rebalance transcriptional output between the autosomes and the X chromosome, genes (or a subset of dosage sensitive genes [2]) on the single X in males were upregulated [3]. Carry through of X chromosome upregulation to females would result in functional X tetrasomy (i.e. 4X: 2A gene dosage), which was subsequently balanced by transcriptional silencing of one X in the somatic cells of females – called X chromosome inactivation (XCI) [4].

During early embryogenesis, in eutherian females, the X inactive specific transcript (*XIST*) is expressed from the X inactivation center (*Xic*) on the future inactive X chromosome (Xi). *XIST*, a long non-coding RNA (lncRNA), coats the X chromosome *in cis* and triggers a cascade of epigenetic events that stably represses transcription of most X loci [5] (reviewed in [6]). In human, genes in older regions of the X chromosome are more likely to be inactivated, whereas genes located in the evolutionarily younger regions of the X chromosome are more likely to be expressed from the Xi (the escaper genes) [7,8]. The choice of which X chromosome (maternal or paternal) becomes inactivate is random [4,9-12]. Although some properties of XCI mechanism vary between species (like timing of XCI initiation [12]), the general epigenetic profile of the Xi appears to be well conserved across eutherian mammals [9,13-15].

Eutherians diverged from the marsupial lineage ~180 million years ago (MYA). The marsupial X is homologous to the long arm and proximal short arm of the human X (denoted as the X conserved region XCR [16]). Like eutherian mammals, one of the two Xs in marsupials is inactivated [17], and although some epigenetic features of the Xi are conserved between clades [18], there are also considerable differences [19,20]. The region homologous to the eutherian *Xic* has been disrupted on the marsupial X chromosome [21-23] and there is no *XIST* homologue [24]. Instead, the lncRNA gene *RSX* (RNA on the silent X) appears to be involved in marsupials XCI [25]. Like *XIST*, the *RSX* transcript is exclusively expressed from the Xi and coats it *in cis* [25].

Early studies of allozyme variants and somatic cell hybrids of four genes in different marsupial species (*G6PD*, *PGK1*, *GLA* and *HPRT* [26-30]) proposed that marsupial XCI was incomplete and variable between tissues and species (reviewed in [17]). Additionally, the results showed that there was imprinted inactivation of the paternally derived X chromosome (as has been described for extraembryonic tissues of rodents and cow [31-33]). A recent genome-wide study in opossum (*Monodelphis domestica*) fetal brain and extraembryonic tissues reported complete paternal inactivation for most (86%) genes on the X chromosome, and escape from XCI for 14% of genes [34]. This level of escape is comparable to that reported for the whole human X (15%). However, it is higher than the level of escape on both the mouse X (3%) [35], and the regions of the human X homologous to the marsupial X (6.5%) [7,35].

Comparison of XCI between different tissues and species offers new insights to its mechanism and evolution. In this study we used RNA-Seq data from a captive bred tammar wallaby family (i.e father, mother and daughter), and genome sequencing from the father and the mother [36], to determine the parent of origin of expressed alleles on the daughter’s X chromosome. We confirm paternal inactivation on the tammar wallaby X chromosome, but observed more escape from inactivation than previously reported for human, mouse and opossum.

## Results and discussion

Imprinted inactivation of the paternal X chromosome in marsupials was hypothesised more than 20 years ago based on evidence from four genes in six different marsupial species [17]. Consistent with this, a more recent study [34] confirmed preferential paternal inactivation as a chromosome-wide phenomenon in opossum brain and extraembryonic tissues. Here we analysed expression of X-linked genes in liver and blood from an Australian marsupial, the tammar wallaby. We sequenced polyadenylated RNA (RNA-sequencing) from a father and mother blood sample, along with a liver sample from the daughter. To overcome the limitation of some alleles not being present in the mother transcriptome due to silencing via XCI, we also used DNA-sequencing data available from the father and the mother [36].

### Mapping results and SNP calling

An average of ~50 million (M) reads were obtained for each of the three samples, of which approximately 26 M, 20 M and 30 M were uniquely mapped for father, mother and daughter respectively (Additional file 1). In total 44,095 SNPs were called between the wallaby reference genome and our three samples. 68% of SNPs were shared between the three individuals (due to being a captive bred colony), so were non-informative when determining parent-of-origin of SNPs in the daughter. A total of 356 SNPs (~1.5% of total) called (in at least one sample) against the reference genome were assigned to the X chromosome (Additional file 2). SNPs that were associated with genes (or scaffolds with genes) that did not have a 1:1 orthologue in human and/or opossum, plus those that we were unable to anchor to the X chromosome, were not included in the analysis (see Methods). Some reported SNPs were located outside wallaby annotated genes and/or exons, likely due to the poor assembly and annotation of the tammar wallaby genome, and were still considered in the analysis.

### Assessing allele-specific expression

We calculated relative expression of each allele for heterozygous biallelically expressed SNPs in the daughter’s liver and the mother’s blood (as performed by Wang *et al.* [34]). In addition, we also included SNPs that were confidently called as heterozygous in daughter (following Mendelian inheritance based on parental genotype), but that were monoallelically expressed (i.e. SNPs that appeared as homozygous in the transcriptome). As such, we could calculate relative expression of each allele for both biallelically and monoallelically expressed heterozygous SNPs.

We observed apparent biallelic expression from the X chromosome in the father, likely because Y-derived reads from the male were reported as best mapped to the X chromosome. This is not surprising since a female individual was sequenced and Y sequence is absent in the tammar wallaby genome assembly [37]. To date, 17 genes have been identified on the tammar wallaby Y chromosome, almost all of which have an X-linked copy [36,38]. We identified 9 X genes with heterozygous SNPs in male including *UBA1*X and *ATRX*, both of which have a known copy on the wallaby Y chromosome (*UBE1Y* and *ATRY*) [38] (Additional file 3). The remaining genes on the X with apparent biallelic expression in males could represent unidentified homologies between the X and Y in tammar wallaby, or multi copy genes on the X; these SNPs were all removed from the analysis.

SNPs were detected for 200 X-linked loci in daughter (Additional file 2); based on parental transcriptome and genome, as well as the daughter transcriptome, we could infer the daughter’s genotype and determine the parent-of-origin of for 42 SNPs in 34 loci (Additional file 4). Twenty-seven SNPs (representing 23 different loci; 68%) had complete monoallelic expression, all of which were exclusively expressed from the maternal allele (i.e. paternal XCI; Additional file 4).

The remaining 15 SNPs (representing 11 loci; 32%) had biallelic expression and were considered escapers (>10% of reads from the lowly expressed allele); the proportion of escape from XCI that we observed in wallaby was considerably higher than that previously reported for human (15%), mouse (3%) and opossum (14%) [7,34,35]. Wang *et al* [34] focused on heterozygous biallelically expressed SNPs, which could potentially underestimate the proportion of inactive loci because heterozygous SNPs expressed from a single allele would be overlooked. When analysing only heterozygous biallelically expressed SNPs on the X, we observed that all loci (29 out of 29) escaped X inactivation in liver (Additional file 2). In addition, we examined heterozygous biallelically expressed X-borne SNPs in the mother’s blood and observed that 2 out of 19 loci escaped inactivation (Additional file 2). This frequency is much higher than the reported for opossum brain and extraembryonic tissues using equivalent methods (14% and 18% respectively).

Average expression from the paternal allele of SNPs that escape XCI was 46% (Additional file 4), whereas in opossum expression from the paternal allele (Xi) is significantly lower than the maternal allele (average of 28%) for most escaper genes [34]. Although different tissues were studied (liver and blood versus fibroblast, brain and extraembryonic tissues), this pronounced escape is likely a reflection of the relative “leakiness” of the tammar wallaby XCI system, which was also observed in tammar wallaby cultured fibroblasts [39].

Generally, SNPs within the same gene or in nearby regions exhibited very similar allelic bias, with the exception of three SNPs mapped to Scaffold28735: two SNPs (X-SNP-I and X-SNP-E) were biallelically expressed, and the nearby SNP (X-SNP-V) ~1.3 kb down stream had paternal inactivation (Additional file 4).

### Inactive status of annotated genes

To determine inactivation status for tammar wallaby X genes we examined only the SNPs within annotated genes. Complete paternal inactivation was observed for ten genes (Table 1) and unbiased biallelic expression for seven genes (Table 1). SNPs in *EBP*, *RPS4X* and *VAMP7* were validated as heterozygous in daughter by conventional sequencing (Additional file 5).

**Table 1:** **Expression status for the 17 wallaby annotated X**-**linked genes for which parent**-**of**-**origin could be determined**

**Ensembl ID**	**Gene name**	**Coverage**	**Father**	**Mother**	**Inactive status**	**Escapee**	**Inactive**
*ENSMEUG00000006054*	*NAA10*	8	62%	38%	Biallelic	-	-
*ENSMEUG00000012279*	*SMC6*	39	54%	46%	Biallelic	-	-
*ENSMEUG00000005589*	*CHM*	6	33%	67%	Biallelic	-	-
*ENSMEUG00000008176*	*HCFC1*	10	30%	70%	Biallelic	*Mdo*/*Hsa*	*Mmu*
*ENSMEUG00000004133*	*HTATSF1*	10	40%	60%	Biallelic	-	*Hsa*/*Mmu*
*ENSMEUG00000002761*	*MECP2*	7	57%	43%	Biallelic	*Mdo*	*Hsa*/*Mmu*
*ENSMEUG00000015172*	*VAMP7*	30	40%	60%	Biallelic	-	-
*ENSMEUG00000015485*	*RAS11LC*	30	0%	100%	PI	-	-
*ENSMEUG00000015892*	*RBMX*	6	0%	100%	PI	-	-
*ENSMEUG00000002871*	*EBP*	50	0%	100%	PI	-	-
*ENSMEUG00000012762*	*LAS1L*	5	0%	100%	PI	*Mdo*/*Hsa*	*Mmu*
*ENSMEUG00000007878*	*OPHN1*	7	0%	100%	PI	-	*Mdo*/*Hsa*/*Mmu*
*ENSMEUG00000011740*	*PFKFB1*	26	0%	100%	PI	-	*Hsa*
*ENSMEUG00000007969*	*RPS4X*	23	0%	100%	PI	*Mdo*/*Hsa*	*Mmu*
*ENSMEUG00000003387*	*HAUS7*	20	0%	100%	PI	-	*Mmu*
*ENSMEUG00000003603*	-	6	0%	100%	PI	-	-
*ENSMEUG00000000692*	*TSPAN6*	5	0%	100%	PI	*Hsa*	*Mmu*

PI denotes paternal inactivation. Escapee column denotes genes that are confirmed as escaping X inactivation in other mammalian species; inactive column denotes genes that are confirmed as inactive in other mammalian species (*Mdo*: *Monodelphis domestica* [34]; *Hsa*: *Homo sapiens* [7]; *Mmu*: *Mus musculus* [35]; -: not information available).

Finally, we attempted to determine parent of origin for expression of *RSX* (which is expressed exclusively from the inactive X chromosome [25]) to better elucidate which X chromosome was inactivated. In daughter no biallelic expression (heterozygous SNPs in the transcriptome data) of *RSX* was observed, and because there was no coverage of RSX in either of the parental genomes the daughter’s genotype could not be inferred. Genotyping (via Sanger sequencing; data not shown) 4.4 kb of the non-repetitive regions of RSX in each individual revealed no heterozygous SNPs. It was, therefore, impossible to determine whether *RSX* had monoallelic expression from the paternal allele in the daughter.

Our previous work, using RNA-FISH to conduct single-cell expression assays in tammar wallaby fibroblast nuclei, provided a similar result to this study where all loci tested escaped XCI to some degree [39]. Only two genes sampled in the present study were assayed previously: 1) *MECP2* was expressed from both X chromosomes in 41% of female nuclei, and in the present study we observed nearly equal expression from the paternal and maternal alleles (Table 1). And 2) *HCFC1* was expressed from both X chromosomes in <10% nuclei (considered inactive), whereas in the present study it was expressed at 30% from paternal allele and 70% from the maternal allele (Table 1). Contrary to our findings, none of the genes studied in [39] completely escaped XCI (i.e. expressed from both X chromosomes in all nuclei), a discrepancy likely due to the different cell types studied.

### Inactivation does not correlate with location on the X in marsupials

**Figure 1:**
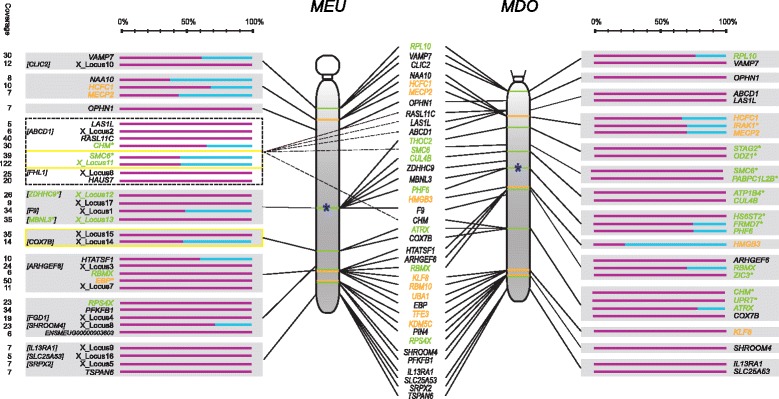
**Parent of origin expression status of X**-**linked loci on the wallaby and opossum X chromosome.** The position of SNPs on the wallaby X chromosome was predicted using the physical map [40] and opossum sytenic blocks with 1:1 orthologous genes. SNPs in wallaby intergenic regions are labelled as X_Locus 1-17, with the name of the neighbouring gene in brackets. SNPs for which position could not be resolved in wallaby are shown in dotted lines. Read depth at each position is given in the ‘Coverage’ column. Yellow boxes identify SNPs within the same scaffold. Orange represents genes in the newest X stratum in marsupials, genes highlighted in green are in the oldest stratum, genes highlighted in black could not be assigned a stratum, and the stars (*) beside gene names denote predicted new or old genes (see Methods). Parent of origin expression of the opossum genes were obtained from [34]. Bars represent the percentage of reads derived from the maternal (pink) or paternal (blue) allele. The dark blue stars are the predicted locations of RSX in each species.

Biallelic expression ratios of close to 1:1 were observed along the length of the X chromosome with no apparent correlation between inactivation and locus location, or correlation with proximity to *RSX* (Figure 1). This is in stark contrast to human, where X-linked genes located in newer strata are more likely to escape inactivation than genes in the older strata. In the human XCR (homologous to the marsupial X) 30 of 462 (6.5%) sampled genes escape inactivation [7]. This is significantly fewer (Fisher’s exact; p = 6.2e-06) than the expected number of escapee genes (69 of 462) should the XCR have the same frequency of escape from inactivation as the whole X.

Two evolutionary strata were recently identified on the marsupial X (here called ‘old’ and ‘new’ [36]), into which X-linked loci were binned when possible (see Methods). We did not observe a correlation with escape from inactivation and location in either of the two marsupial X evolutionary strata [36]. Seven of the 34 wallaby X loci sampled could be classified as members of the oldest X stratum, with three escaping XCI. Although the number of genes sampled in the old stratum was low, the frequency of escape remained similar to the frequency of escape on the whole X (32%). Likewise, of the three loci assigned to the newer tammar wallaby X stratum, two escaped inactivation.

After examination of previously published opossum X inactivation data ([34]; see Methods) just five genes could be assigned to the newer stratum, of which four escaped inactivation. However, 52 genes with known XCI status were placed in the oldest marsupial X stratum, of which five escaped XCI. In contrast to the different inactivation levels between human X strata, the number of escapee genes in the opossum old X stratum was not statistically different (Fisher’s exact; p = 0.7602) from the expected number of escapee genes (7 of 52; calculated from the whole X frequency of escape: 24 of 176).

## Conclusions

Our results demonstrate that chromosome wide inactivation of the paternally inherited X is common to both Australian and American marsupials. Also, in stark contrast to human where just 6.5% of genes located in the two oldest X chromosome evolutionary strata escape inactivation and 40.7% escape inactivation in the three youngest strata, we show that a higher probability of inactivation does not correlate with location in the oldest marsupial X stratum.

## Methods

### Sample collection

Samples were collected and held under the Australian National University’s Animal Experimentation Ethics Committee proposal number A2011/57, and the CSIRO Sustainable Ecosystems Animal Ethics Committee application number 10-02. Blood samples were obtained from the mother and the father, and liver tissue sample was obtained from a 12 week old female pouch young and snap frozen in liquid nitrogen.

### RNA extraction and sequencing

Total RNA from parental blood was extracted from 400 uL starting material using the RiboPure Blood™ Kit (Ambion) following manufacturer’s instructions. RNA from liver was extracted using the GenElute® Total Mammalian RNA Extraction Kit (Sigma-Aldrich) following the manufacturer’s guidelines. RNA integrity (RIN) and quantity was checked on an Agilent 2100 Bioanalyzer (Agilent Technologies). RNA (RIN >7) was shipped in RNAstable® storage tubes (Biometrica). A single run for each sample was performed at the Beijing Genomics Institute (BGI), Tai Po, Hong Kong. Poly(A) mRNA was isolated utilizing oligo(dT) beads, fragmented and random hexamer-primers were used to synthesize single-strand cDNA, followed by a second round of cDNA synthesis. Fragments were purified and concentrated with the QiaQuick® PCR Extraction Kit, eluted with EB buffer for end repair followed by addition of poly(A). Resulting fragments were ligated with sequencing adaptors, size-selected and single end 90 bp reads were generated using the Illumina HiSeq™ 2000 platform. These sequences are available at the NCBI Sequence Read Archive (SRA) (http://www.ncbi.nlm.nih.gov/sra; accession number: PRJNA258238).

### Read mapping

Raw reads were processed using the web-based Galaxy server [41] (https://usegalaxy.org). Read quality was assessed using the FastQC software available at the Galaxy server (v0.52) and processed with the FASTX-Toolkit (v.0.0.13). Reads were mapped using TopHat v1.1.2 [42] to the wallaby genome release 74 (MacEug1.0.74) (ftp://ftp.ensembl.org/pub/release-74/fasta/macropus_eugenii/dna/), allowing a maximum of one mismatch and reporting only uniquely aligned reads. Post-mapping processing and duplicate removal was done with SAMTools (v.0.1.18) [43]. Reads were visualized using Integrative Genome Viewer (IGV) [44].

### Single nucleotide polymorphism (SNP) calling and filtering

For SNP calling, SAMTools [43] and VarScan (v2.3.5) [45] was utilised, high coverage bases (≥5 at each SNP position) were reported, and were manually curated. Heterozygous SNPs with a single read supporting either allele were compared to SNPs in the vicinity (i.e. SNPs in the same gene; or in a ~20 kb window for SNPs in intergenic regions) and were ignored if at least three neighbour SNPs were homozygous and no heterozygous SNPs were present in the vicinity.

### Parent of origin

Parent of origin for each allele in the daughter was determined by comparing each SNP to the same position in each parent where information was available from the transcriptome (from this study) and/or genome [36], with three possible outcomes: complete inactivation, ambiguous or biallelic expression (Additional file 6). For SNPs with biallelic expression, we quantified allele-specific expression as the ratio of the number of reads supporting a particular allele divided by the total coverage at that position; SNPs were considered escapers if more than 10% of reads support the lowly expressed allele [7].

### Assigning SNPs to chromosomes and annotated gene exons

Wallaby annotated protein-coding genes with 1:1 human orthologues were obtained from Ensembl74. Gene scaffolds in the tammar wallaby were assigned to the X chromosome when at least 1:1 orthologue on the human XCR and/or the opossum X was observed. Genes were assigned as autosomal if there was 1:1 orthologue on the remainder of human X chromosome, or on human autosomes. SNPs in exonic regions were annotated based on exon genomic coordinates available from Ensembl74. For X-assigned SNPs mapped to intergenic regions in X scaffolds, a 1000 bp window (500 bp upstream and 500 bp downstream) was search with BLAST (discontiguous megablast) against the NCBI nucleotide collection, to identify potential unannotated tammar wallaby genes that were orthologues to known human or opossum X-linked genes.

### Assigning loci to X chromosome strata

Opossum X-linked loci were classified as ‘old’ or ‘new’ genes according to the strata information in Cortez *et al.* [36]. Loci that mapped between two X genes in the old stratum were considered old loci. Similarly, X-linked loci located between two new genes were considered to be in the new stratum. All loci located between an old and a new gene were excluded from the strata analysis. Conserved syntenic blocks between opossum and wallaby [40] were used to assign genes to the new or old strata in tammar wallaby.

### RSX

Homology to the opossum *RSX* was previously reported for three wallaby ESTs: *EX198340.1*, *FY484131.3* and *FY484132.2* [25], all of which map to a ~2 kb region at the end of wallaby Scaffold61551. To detect further regions of homology, the *RSX* complete sequence (X:35,605,415-35,651,609; coordinates from [25]) and individual exons [25] were searched for (using BLAST) in the wallaby reference genome (MacEug1.0.70).

### PCR and DNA sequencing

The DNeasy® Blood and Tissue Kit (Qiagen) were used to extract genomic DNA from each individual, following the manufacturer’s instructions. Primers were designed using Primer3 [46] (Additional file 7). PCR reactions were performed using 100 ng/uL template DNA, MyTaq™ Red Mix (Bioline) and 1 pmol of primers, with initial denaturation at 94°C for 2 minutes, followed by 36 cycles of: 30 seconds at 94°C, 30 seconds at 50°C-65°C, 60 seconds at 72°C; then 72°C for 10 minutes. Resulting products were separated and purified using the PureLink® Kit (Invitrogen). DNA sequencing was performed by the Biomolecular Resource Facilty (The Australian National University), or the Ramaciotti Centre (University of New South Wales). Sequence trace files were visualised using 4 Peaks software from Nucleobytes (www.nucleobytes.com) and sequence alignments were performed in ClustalX 2.1 with default parameters.

### Availability of supporting data

Transcriptome sequences generated in this analysis are available in the NCBI Sequence Read Archive (SRA) database, (http://www.ncbi.nlm.nih.gov/sra), with accession number: PRJNA258238 (http://www.ncbi.nlm.nih.gov/bioproject/258238). Genome sequences are available from [36].

## Additional files

Additional file 1:
**Mapping statistics.**
Additional file 2:
**The 23,613 SNPs that were assigned to an autosome or X chromosome based on 1:1 orthology between wallaby and human autosomes, and wallaby and human/opossum X chromosomes.**
Additional file 3:
**X-linked SNPs in father with biallelic expression (concluded from transcriptome or genome) that were removed from the analysis.**
Additional file 4:
**SNPs on the X chromosome for which parent of origin could be determined in the daughter.**
Additional file 5:
**Genotype validation of three X-linked SNPs via conventional Sanger sequencing.**
Additional file 6:
**Criteria (and examples) followed to determine parent-of-origin for X-linked SNPs in the daughter.** Pink represents maternal allele; blue represents paternal allele; red represents unknown origin. The transcriptome is representative of genotype for X-linked SNPs in the father. Additional file 7:
**Primers used to amplify regions spanning SNPs selected for genotype validation, and amplify random regions in the** ***RSX*** **transcript.**

## Abbreviations

XCI: X chromosome inactivation
XIST: X inactive specific transcript
Xic: X inactive centre
Xi: inactive X chromosome
MYA: Million years ago
XCR: X conserved region
RSX: RNA-on the silent X
SNP: Single nucleotide polymorphism
M: Million
