# Supplementary material for: Paternal X inactivation does not correlate with X chromosome evolutionary strata in marsupials
Source: BMC Evol Biol. 2014 Dec 24;14:267. doi: 10.1186/s12862-014-0267-z (PMC4302592; doi:10.1186/s12862-014-0267-z)
Supplement: Additional file 6: — Criteria (and examples) followed to determine parent-of-origin for X-linked SNPs in the daughter. Pink represents maternal allele; blue represents paternal allele; red represents unknown origin. The transcriptome is representative of genotype for X-linked SNPs in the father. [file 12862_2014_267_MOESM6_ESM.pdf]

**Cases when neither origin of the active allele nor level of expression from each parental allele could be determined (ambiguous):**		
*a) when all three samples (or at least the daughter and a parent) are homozygous and bearing the same allele in **transcriptome** or **genome**.*		
A C T G A T C G	A C T G A T C G	A C T G A T C G
A C T G A T C G	- - - - - - - - - - - - -	A C T G A T C G
- - - - - - - - - - - -	A C T G A T C G	A C T G A T C G
*b) both parental SNPs are homozygous in **transcriptome**, but different to each other, and the daughter homozygous, apparently only expressing the paternal allele. However, the mother could have an allele (A in this case), which is also silenced via XCI.*		
A C T G A T C G	A C T G C T C G	A C T G A T C G
*c) in the cases when the father is not sampled and the mother is homozygous in **transcriptome**, since the mother could have a repressed allele via XCI.*		
- - - - - - - - - - - -	A C T G A T C G	A C T G C T C G
- - - - - - - - - - - -	A C T G A T C G	A C T G A/C T C G
*d) if the father is not sampled and the mother is heterozygous in **transcriptome** or **genome**.*		
- - - - - - - - - - - -	A C T G A/C T C G	A C T G A/C T C G
- - - - - - - - - - - -	A C T G A/C T C G	A C T G A T C G
*e) the mother is heterozygous in **transcriptome** or **genome** and the daughter is homozygous, bearing the paternal allele (A) in **transcriptome** or **genome**; however the daughter could have obtained the allele (A) from both parents and could be expressing both or only one.*		
A C T G A T C G	A C T G A/C T C G	A C T G A T C G
**When biallelic expression was observed in the daughter, we could determine parent-of-origin when:**		
*f) the father is homozygous (**transcriptome** or **genome**), the mother heterozygous (**transcriptome** or **genome**) and the daughter is heterozygous. In this case, the daughter obtained one allele (A) from the father and the second (C) from the mother.*		
A C T G A T C G	A C T G A/C T C G	A C T G A/C T C G
*g) both parental SNPs are homozygous (**transcriptome** or **genome**), but different to each other, and the daughter is heterozygous.*		
A C T G A T C G	A C T G C T C G	A C T G A/C T C G
*h) one of the alleles observed in the daughter is not present in either parent (**transcriptome**). Because we are certain of the father's SNP (A in this example), we can assume the mother is heterozygous at the position but the alternative allele is silenced through XCI (C in this example), thus, the daughter obtained the alternative allele (C) from the mother.*		
A C T G A T C G	A C T G A T C G	A C T G A/C T C G
*i) the father is homozygous (**transcriptome** or **genome**) and the daughter heterozygous.*		
A C T G A T C G	- - - - - - - - - - - -	A C T G A/C T C G
*j) the father is not sampled, the mother is homozygous in **genome** and the daughter is heterozygous.*		
- - - - - - - - - - - -	A C T G A T C G	A C T G C/A T C G
**In the cases when monoallelic expression was observed in the daughter, parent-of-origin could be determined when:**		
*k) one of the alleles observed in the daughter is not present in either parent (**transcriptome** or **low coverage in mother genome**). Because we are certain of the father's SNP (A in this example), we can assume the mother is heterozygous at the position but the alternative allele is silenced through XCI (C in this example). The daughter obtained the alternative allele (C) from the mother (paternal inactivation).*		
A C T G A T C G	A C T G A T C G	A C T G C T C G
*l) both parental SNPs are homozygous (**transcriptome** or **genome**), but different to each other, and the daughter is homozygous expressing the maternal allele (C). Since we are certain the father is homozygous (A in this case), the daughter is repressing the allele from the father (A) and only expressing the maternal allele (C) (paternal inactivation).*		
A C T G A T C G	A C T G C T C G	A C T G C T C G
*m) the father is homozygous (**transcriptome** or **genome**) and the daughter is homozygous, but different to each other, with the mother being heterozygous (**transcriptome** or **genome**); the daughter obtained the allele from the mother (C) and is repressing the allele from father (A) (paternal inactivation).*		
A C T G A T C G	A C T G A/C T C G	A C T G C T C G
*n) the father is homozygous (**transcriptome** or **genome**) same as the daughter but bearing different alleles, and the mother is not sampled. In which case the daughter obtained an A from the father and the expressed allele (C) from the mother (paternal inactivation).*		
A C T G A T C G	- - - - - - - - - - - -	A C T G C T C G
*o) both parental SNPs are homozygous in **genome**, but different to each other, and the daughter homozygous (maternal inactivation).*		
A C T G A T C G	A C T G C T C G	A C T G A T C G
*p) in the case when the father is not sampled and the mother is homozygous in **genome** and daughter is homozygous (paternal inactivation).*		
- - - - - - - - - - - -	A C T G A T C G	A C T G C T C G
